# Do stricter criteria for intraoperative parathyroid hormone monitoring reduce the risk of persistence or reoperation in primary hyperparathyroidism? A receiver operating characteristic analysis

**DOI:** 10.1007/s00423-025-03796-4

**Published:** 2025-07-10

**Authors:** Henning Wendelin Wolf, Sara Canovi, Christian Andreas Nebiker

**Affiliations:** 1 Department of General Surgery, Cantonal Hospital of Aarau, Aarau, Switzerland; 2 Sankt Vinzenz Hospital Rheda-Wiedenbrueck, Rheda-Wiedenbrueck, Germany

**Keywords:** Parathyroidectomy, Parathyroid hormone, Hyperparathyroidism, Parathyroid glands

## Abstract

**Purpose:**

Intraoperative parathyroid hormone (PTH) measurement is a beneficial tool in the surgical management of primary hyperparathyroidism. The expected degree of intraoperative PTH reduction, which guides surgical decision-making, determines the sensitivity and specificity of this test. While stricter criteria may enhance diagnostic accuracy, an optimal threshold has not been conclusively established. The aim of this study was to identify the PTH reduction threshold that provides the highest sensitivity and specificity for achieving biochemical cure.

**Patients and methods:**

A retrospective analysis was conducted on 141 patients who underwent parathyroidectomy for primary hyperparathyroidism, focusing on the intraoperative drop in PTH and surgical success. A receiver operating characteristic analysis was performed to identify the optimal threshold that balances sensitivity and specificity in predicting biochemical cure.

**Results:**

The mean percentage reduction at the end of surgery was 73.93% (SD ± 16.54%) with an overall cure rate of 94%. The area under the curve was 0.73 for a 50% PTH reduction, 0.77 for a 60% reduction, and 0.68 for a 70% reduction.

**Conclusion:**

The optimal balance between sensitivity and specificity was achieved with a 60% intraoperative PTH reduction. Stricter criteria increase sensitivity but may also raise the risk of surgical overtreatment.

## Introduction

Primary hyperparathyroidism (pHPT) is a common endocrine disorder that results in the overproduction of parathyroid hormone (PTH) and subsequent hypercalcemia. Definitive cure can only be achieved by surgery and is recommended for symptomatic elderly and all younger patients [1, 2].

With the increasing preference for open minimally invasive parathyroidectomy (OMIP), the accurate identification of small and/or abnormally located glands has become crucial [3]. In addition to preoperative localization diagnostics, intraoperative identification is essential for surgical success. Near-infrared light-based intraoperative adjuncts and frozen sections can serve as verification tools, but do not rule out multiglandular disease (MGD) [4, 5].

Due to its short half-life, intraoperative PTH (ioPTH) determination is suitable for detecting the presence of hyperfunctional parathyroid tissue and preventing the persistence of pHPT due to multiglandular or ectopic parathyroid adenomas (PA) [6, 7]. However, its usefulness remains controversial among endocrine surgeons [8, 9].

During recent decades, multiple criteria have been published that differ mainly in the timing of blood sampling and the intraoperative parathyroid hormone decrease that is considered positive. According to the widely accepted Miami criteria, PTH samples are obtained before skin incision and/or before excision of the suspected gland(s). A ≥50% ioPTH decrease from the highest pre-excision value, measured ten minutes after the removal of the suspected PA, indicates a successful operation [10, 11].

To consider “PTH spikes” resulting from intraoperative manipulation of the PAs, it may be useful to measure a baseline PTH immediately before and a post-excision PTH 10 min after resection of the parathyroid adenoma [12, 13].

An ioPTH decline exceeding the threshold defined by the Miami criteria may enhance the sensitivity and possibly the specificity of the test [14].

This study aimed to assess whether a greater ioPTH reduction could enhance the sensitivity and specificity for predicting biochemical cure by identifying the optimal reduction threshold.

## Patients and methods

### Patients

Patients with sporadic pHPT who underwent focused parathyroidectomy from January 2016 to December 2022 were included in this study. The diagnosis of pHPT was established by our Department of Endocrinology based on clinical, laboratory, and imaging findings, as well as by external specialists who referred patients for surgical treatment. Preoperative localization of the pathological parathyroid gland was determined in all patients by high-resolution ultrasonography of the neck and 99mTc-methoxyisobutyl isonitrile (99mTc-MIBI) scintigraphy. In case of an inconclusive imaging, a Choline Positron Emission Tomography/Computed Tomography (PET/CT) was performed. The operations were conducted by three surgeons accredited in Endocrine Surgery at the Department of Visceral Surgery, Cantonal hospital of Aarau (Aarau, Switzerland). The analysis of this data was retrospective. This study was conducted in accordance with the Declaration of Helsinki (as revised in 2013) and general consent was obtained from all included patients.

Exclusion criteria were patients under 18 years, those with secondary hyperparathyroidism or congenital syndromes related to hyperparathyroidism, individuals with a history of parathyroidectomy, suspected renal hyperparathyroidism, and those lacking patient consent. The approval of the local ethics committee was obtained (ethics committee of northwestern and central Switzerland; BASEC-ID 2023 − 01479).

### Parathyroid operation

All operations were performed via Kocher`s incision. Based on the preoperative localization diagnostics, the suspected PA was exposed. A first single baseline plasma ioPTH sample was then obtained prior to excision of the suspected PA. The second sample was obtained 10 min after the adenoma had been removed. According to our institutional standard which is based on the findings reported by Lupoli et al., a 70% decrease was considered indicative of the removal of all hyperfunctioning parathyroid tissue (13). In the case of an inadequate ioPTH decrease, exploration was continued. If a second adenoma was identified, ioPTH was repeated 10 min after its removal.

In one case, the operation was discontinued despite an inadequate drop in ioPTH due to the patient’s refusal to consent to further exploration. When the exploration of all four parathyroid glands was unsuccessful, the operation was stopped, and additional imaging with choline PET/CT was performed.

### Follow up

Albumin-corrected serum calcium and PTH levels were monitored at postoperative day one. Further clinical and biochemical follow up was conducted by the attending endocrinologist. Cure was defined as normocalemia (2.15–2.60 mmol/L) for at least six months postoperatively. Recurrence was therefore defined as elevated PTH (> 68.0 ng/L) and calcium (> 2.6 mmol/L) at the earliest 6 months after initially successful surgery, while persistence was defined as elevated PTH and calcium levels for at least six months postoperatively.

### Statistical analysis

Correlation between ioPTH reduction and reoperation/persistence rate were calculated using chi-squared test or Fisher`s exact test. A receiver operating curve (ROC) analysis was performed to assess the predictive value of intraoperative PTH reduction for persistent disease. The sensitivity and specificity values were calculated for each percentage reduction in ioPTH levels. A true positive was defined as the accurate prediction of persistent disease. The ROC analysis was then used to determine the IOPTH percent drop with the best performance (sensitivity and specificity) in predicting persistence. The Youden Index is a measure of ‘informedness’ that allows generalization of the dichotomous outcome for the test being examined. In this case, it is an indicator of how informative a particular PTH reduction value may be for predicting a curative operation. The odds ratios (OR) for the different cut off (60% and 70%) were analyzed to quantify the association between intraoperative PTH reduction and the likelihood of persistent disease and reoperation, providing a measure of how different PTH reduction thresholds correlate with the probability of disease persistence. The level of significance was taken to be p < 0.05. Statistical analysis was performed using IBM SPSS (Statistical Product and Services Solutions, version 24 for Windows).

## Results

### Preoperative patients and imaging results

**Fig. 1:**
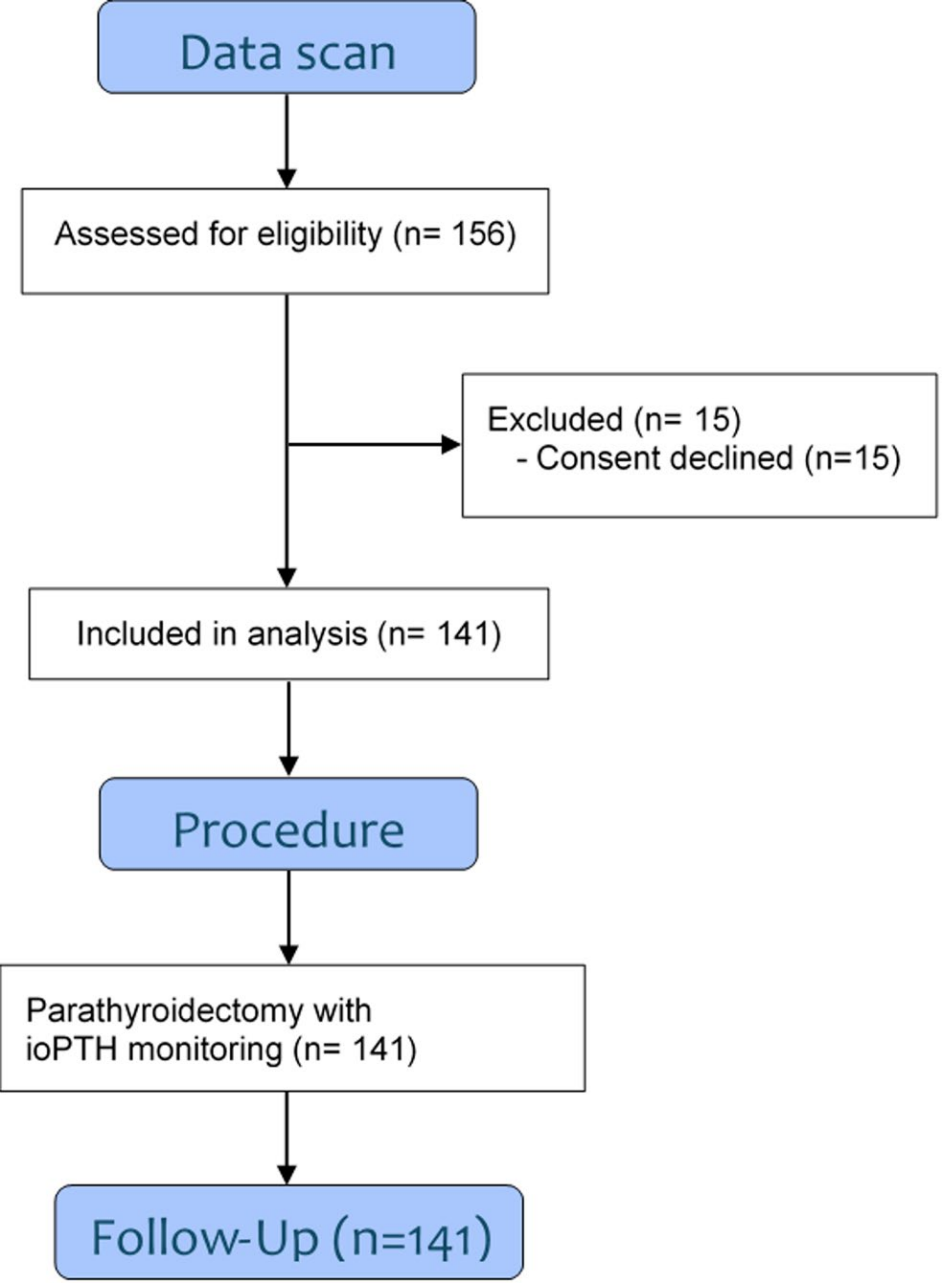
CONSORT 2010 flow diagram

Of 163 consecutive patients with sporadic pHPT who underwent parathyroidectomy, 141 were included in this study. 7 patients were excluded due to revision surgery, and in 15 cases, consent was refused (Fig. 1). Detailed patient characteristics are presented in Table 1. In 139 patients, neck ultrasound was conducted as the primary imaging modality, with 99mTc-MIBI scintigraphy serving as the secondary imaging modality in 130. In 38 patients, choline PET/CT served as a tertiary imaging modality, and in 11 cases the secondary imaging modality was chosen due to radiation protection or patient refusal.

### Parathyroid surgery

121 patients underwent OMIP, while planned bilateral neck exploration (BNE) was conducted in 7 cases. Conversion from OMIP to BNE was necessary in 13 patients. In 17 patients, parathyroidectomy was combined with thyroid resection for multinodular goiter (8 patients) or presumed intrathyroidal PAs (9 patients). There were no cases requiring conversion to sternotomy or revision surgery for postoperative hemorrhage.

### Intraoperative PTH levels

The mean baseline plasma PTH level of the entire patient cohort prior to excision was 296.40 ng/L (SD ± 306 ng/L). Ten minutes after resection, the mean ioPTH level was 64.65 ng/L (SD ± 64 ng/l). The mean percentage reduction in ioPTH at the end of surgery was 73.93% (SD ± 16.54%).

A total of 103 patients (73%) demonstrated an ioPTH drop of > 70%, indicating the successful excision of all hyperfunctioning parathyroid tissue. In 29 cases (20,5%) there was a borderline ioPTH reduction (50–70%). In cases where the adenoma was macroscopically clear, a frozen section examination was performed. When hyperfunctional parathyroid tissue was histologically confirmed, the surgical procedure was terminated without further exploration.

In 13 cases (9.22%), inadequate ioPTH reduction and a macroscopically normal parathyroid gland led to bilateral neck exploration. In 7 of the 13 cases a second adenoma was identified, while in 6 patients further exploration failed to identify additional hyperfunctional parathyroid tissue. In these cases, surgery was terminated, and further post-operative imaging was ordered (Table 1).

**Table 1:** Patients characteristics and laboratory findings

Gender	
Male, n (%)	36 (25.5)
Female, n (%)	105 (74.5)
Age at operation	
Mean ± SD	62,58 ± 13.9
Procedure	
Unilateral exploration, n (%)	121 (85,8)
Planned bilateral exploration, n (%)	7 (5)
Unplanned bilateral exploration, n (%)	13 (9.22)
Additional thyroid resection, n (%)	17 (12.1)
Mean calcium ± SD (mmol/L)	
Preoperative	2.75 ± 0.20
Post excision	2.41 ± 0.12
Mean parathyroid hormone ± SD (ng/L)	
Preoperative	215.23 ± 212.64
Pre excision	296.4 ± 306
Post excision	64.65 ± 64
POD 1	41.8 ± 37.07
Mean relative Parathyroid hormone reduction in % (± SD)	
Pre excision to post excision	73.93 ± 16.54

*POD *postoperative day, *SD *standard derivation

### Follow up

**Fig. 2:**
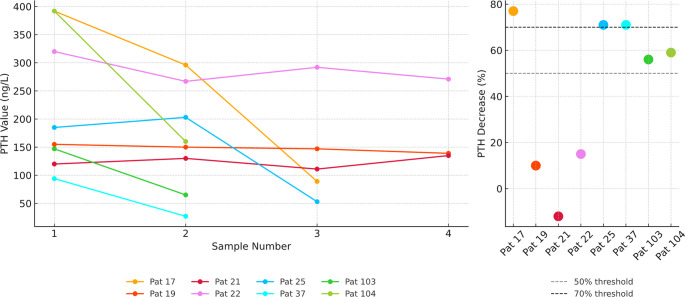
Intraoperative PTH values and decrease in patients with persistent disease

Postoperatively, the values were measured at least once during the first three months after resection and again at six months after the initial surgery. Six months following the initial procedure, 133 patients exhibited normocalcemia and were therefore classified as ‘cured.’ Conversely, 8 patients presented with hypercalcemia in two postoperative follow-ups and were thus categorized as having ‘persistent disease’. Among patients with persistent disease, ioPTH reduction ranged from + 12% to −77% (Fig. 2).

### Receiver operating characteristic analysis

**Fig. 3:**
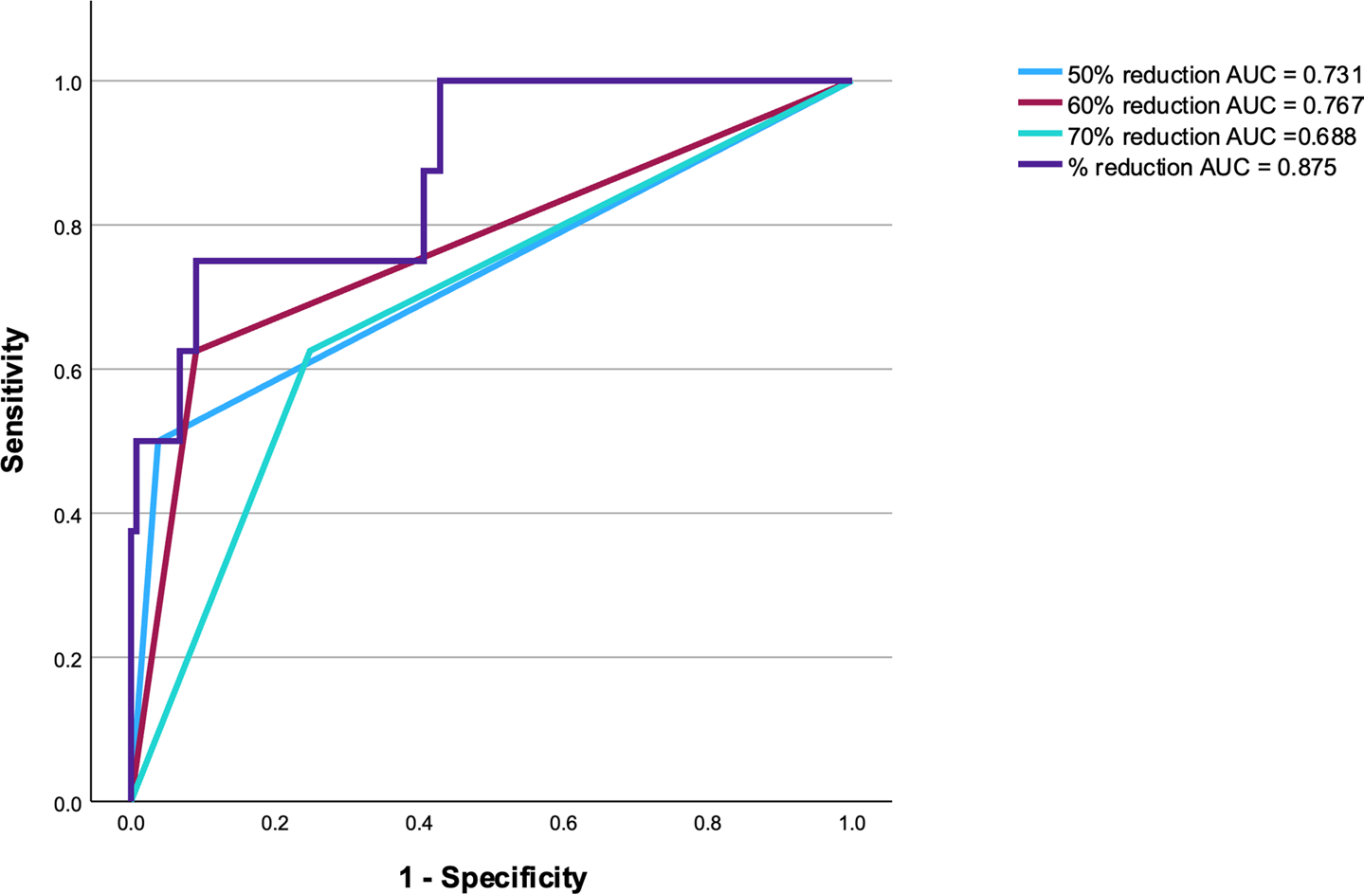
Receiver operating characteristic analysis

The Area Under the Curve (AUC) for the “% reduction” method was 0.875, indicating very good overall diagnostic accuracy. The maximum Youden Index of 0.66 was observed at a threshold of 59.82% reduction, corresponding to a sensitivity of 75% and a specificity of 91% (Fig. 3). This threshold achieves the best trade-off between detecting true positives and minimizing false positives. Thresholds below 59.82% decreased specificity, while higher thresholds reduced sensitivity, increasing the risk of missing cases with persistent disease. The application of a 60% threshold would result in a reduction of false positives (9%) compared to the 24.1% observed under the inhouse 70% threshold scenario, while maintaining the same percentage of false negatives (37.5%). Conversely, if a 50% threshold according to the Miami criterion would be considered, the false positive rate would decrease to 3.8%, however, this would be accompanied by an increase in the false negative rate (50%, Table 2).

**Table 2:** Test performance at different IoPTH reduction scenarios

PTH cut-off (%)	Sensitivity (%)	Specificity (%)	False positives (%)	False negatives (%)
70	75	75	24,08	24,08
60	75	91	9	37,5
50	50	96,2	3,8	50

Inverse relationship between sensitivity and specificity at decreasing PTH cut-offs. PTH, parathyroid hormone; ioPTH, intraoperative parathyroid hormone measurement.

The same ROC analyses were performed for the outcome of reoperation; however, due to the limited number of cases (only five cases of reoperation), the ROC curve did not yield significant results. Notably, in three cases, patients refused reoperation despite persistent disease.

### Risk analysis and clinical implications

**Fig. 4:**
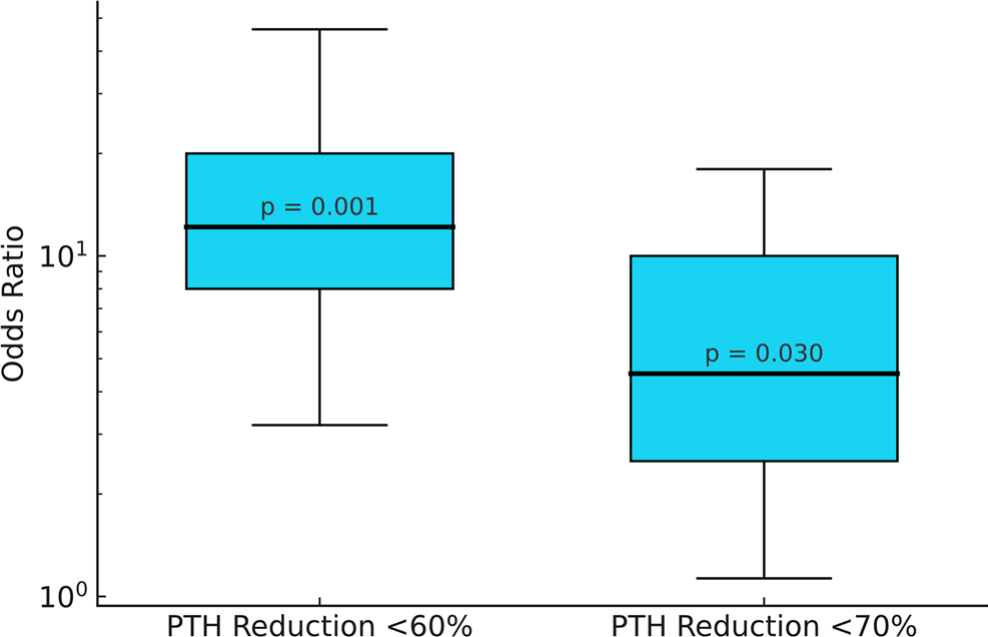
Odds Ratio for persistent disease

In accordance with the findings of the ROC analysis, a risk analysis was conducted to evaluate the association between intraoperative PTH reduction and the likelihood of persistent disease and reoperation. The odds ratio (OR) was calculated for two clinically relevant cut-off values: 60% and 70%. At a PTH reduction below 60%, the OR for persistence was 12.16 (95% CI: 3.19–46.36, *p* = 0.001), indicating a significantly higher risk of persistent disease compared to patients with a reduction above this threshold. Similarly, the OR for reoperation was 10.94 (95% CI: 1.97–60.85, *p* = 0.013), demonstrating that patients who failed to achieve at least a 60% reduction were at a markedly increased risk of requiring further surgical intervention. When applying a 70% PTH reduction threshold, the OR for persistence was lower at 4.52 (95% CI: 1.13–17.99, *p* = 0.03), suggesting that while this cut-off still holds predictive value, its association with persistent disease is weaker than that of the 60% threshold. Furthermore, the 70% cut-off did not result in a statistically significant reduction in reoperation rates, likely due to the small number of cases (Fig. 4).

## Discussion

There remains a lack of consensus regarding the optimal diagnostic criterion for ioPTH monitoring. The optimal cutoff remains uncertain, particularly when balancing sensitivity and specificity.

The only other published study employing ROC analysis to determine the optimal predictive threshold for successful surgery is the one of Foley et al. which encompassed 869 patients [15]. Their study aimed to predict multi-glandular disease (MGD), with their ROC analysis yielding an AUC of 0.69. In contrast, our investigation, which included a smaller cohort of 141 patients, analyzed values taken at the end of the operation, either after the identification and removal of an adenoma or following additional attempts and further explorations. These decisions were influenced by the surgeon’s experience and preoperative localization diagnostics. Our ROC analysis revealed a superior AUC of 0.875, indicating better overall diagnostic accuracy. Foley et al. identified 72% as the optimal threshold for predicting MGD, yielding a sensitivity of 55% and specificity of 76%. Our study, however, focused on identifying the optimal threshold to reduce the risk of persistent disease, and our findings suggest a better-performing threshold of 60% with a sensitivity of 75% and specificity of 91%.

On the other hand, the recently published network meta-analysis by Staibano et al. has provided a comprehensive evaluation of the diagnostic accuracy of various ioPTH monitoring criteria in the context of pHPT [16]. Their findings highlighted the modified Miami criteria, which demonstrated the highest diagnostic OR (79.71) among all assessed criteria. This criterion requires a ≥ 50% decrease in ioPTH levels at least 15 min after excision of all hyperfunctioning parathyroid tissue and optimizes diagnostic accuracy by minimizing unnecessary neck exploration and revision surgery. In contrast with our data, we reached a diagnostic OR of 30 and an accuracy of 0.77%., achieving a high sensitivity of 96.2%, at the cost of reduced specificity (only 50%) (Youden Index 0.47), which in our population meant missing 5 out of 8 cases of persistent disease.

Similarly, a 70% cutoff, as suggested by Lupoli et al., provided a specificity of 75% but did not improve the sensitivity compared to the 60% threshold, potentially leading to overtreatment without additional clinical benefit [14].

The present study identified an overall persistence rate of 5.6%, which is higher than the 6-month persistence rates reported in the literature after minimally invasive parathyroidectomy (MIP), which range from 1 to 5% [17]. Notably, most cases of persistent disease occurred in 2017 (five cases), while from 2018 to 2022, only three events were observed. Excluding the outlier year of 2017, the persistence rate drops to approximately 0.03%. This marked improvement is likely the result of enhancements in preoperative localization techniques and increased surgeon experience with the available methods.

The value of ioPTH may also be influenced by increasingly precise preoperative localization techniques, such as choline PET/CT. While for example Scipioni et al. and Vaghaiwalla et al. postulate that ioPTH can be omitted with accurate preoperative imaging, several studies emphasize the importance of ioPTH in parathyroid surgery even with concordant preoperative imaging studies (ultrasound and scintigraphy or PET/CT) [8, 18–20]. Our experience echoes these findings. We observed that utilizing ioPTH monitoring as part of a broader decision-making framework, encompassing intraoperative findings and the surgeon’s clinical judgment, can help reduce the invasiveness of the procedure while minimizing the risk of persistent disease.

This study has several limitations. It is a retrospective analysis, which inherently introduces the potential for selection bias and limitations in data completeness. Additionally, the surgeries were performed by different surgeons with varying levels of experience. This variability could have influenced both the decision-making process during surgery and the outcomes. In some cases, despite an inadequate PTH drop or inconclusive diagnostic findings, further exploration was not pursued because the patient did not consent to additional procedures. These factors may have affected both diagnostic accuracy and clinical outcomes.

## Conclusion

IoPTH monitoring remains an important adjunct in the surgical management of pHPT. Based on this data analysis, the optimal PTH threshold appears to be approximately 60%, offering a pragmatic balance between minimizing the risk of persistent disease and avoiding unnecessary reoperations. However, a higher test accuracy compared to established methods could not be achieved.

## Data Availability

No datasets were generated or analysed during the current study.
